# Stochastic contagion models without immunity: their long term behaviour and the optimal level of treatment

**DOI:** 10.1007/s10100-018-0526-y

**Published:** 2018-02-07

**Authors:** Raimund M. Kovacevic

**Affiliations:** Institute of Statistics and Mathematical Methods in Economics, Vienna University of Technology, Vienna, Austria

**Keywords:** Contagion, Asymptotic properties, Markov processes, Disease control

## Abstract

In this paper we analyze two stochastic versions of one of the simplest classes of contagion models, namely so-called SIS models. Several formulations of such models, based on stochastic differential equations, have been recently discussed in literature, mainly with a focus on the existence and uniqueness of stationary distributions. With applicability in view, the present paper uses the Fokker–Planck equations related to SIS stochastic differential equations, not only in order to derive basic facts, but also to derive explicit expressions for stationary densities and further characteristics related to the asymptotic behaviour. Two types of models are analyzed here: The first one is a version of the SIS model with external parameter noise and saturated incidence. The second one is based on the Kramers–Moyal approximation of the simple SIS Markov chain model, which leads to a model with scaled additive noise. In both cases we analyze the asymptotic behaviour, which leads to limiting stationary distributions in the first case and limiting quasistationary distributions in the second case. Finally, we use the derived properties for analyzing the decision problem of choosing the cost-optimal level of treatment intensity.

## Introduction

In epidemiology, one of the simplest types of models is given by SIS models. They describe the spread of infectious diseases without immunity, i.e. it is assumed that individuals can be infected multiple times throughout their lives and that no immunity happens after infection. The acronym SIS illustrates these assumptions: it is possible for a susceptible (S) individual to become infected (I), and later on to become susceptible (S) again. Typical examples for diseases that can be modelled in this way—at least in first approximation—are rota-viruses, some sexually transmitted infections and many bacterial infections, see e.g. Hethcote and Yorke (1984) and Brauer et al. (2008).

In the present paper we use the language of epidemiology, e.g. speaking of “infection”, “recovery” or “disease”. However, contagion models play an important role also in various disciplines of social sciences, management science and economics, when the spreading of information or behavior (the “disease”) amongst sub-populations is modelled. Examples for such fields are marketing (e.g. Gould 1970; Mahajan et al. 1993), rumour modeling (e.g. Kandhway and Kuri 2014) or illicit drug dynamics (e.g. Behrens et al. 2002).

We denote the number of infected individuals at time $t\in {\mathbb {R}}$ by *X*(*t*) and the number of susceptible individuals by *Y*(*t*). In the simplest case (without birth and death), the overall population with size *N* is assumed to be constant. Note that because of

1 $$\begin{aligned} N=X(t)+Y(t) \end{aligned}$$

it suffices to model the number of infected, replacing *Y*(*t*) by $N-X(t)$ whenever necessary. Two parameters are relevant: the disease transmission coefficient (or strength of infection) $\beta >0$, leading to a force of infection^1^ (or incidence rate) of $\beta X(t)$, and the recovery rate $\gamma >0$. The force of infection models the rate at which susceptible individuals become infected, while the recovery rate is interpreted as the rate at which infected individuals become susceptible again, which means that $1/\gamma$ is the duration of infection.

### Deterministic SIS model

While we aim at stochastic SIS models in continuous time, deterministic models are most widely used in epidemiology, as well as in the economic and socio-economic context. In particular, all papers cited as examples for applications of contagion models in the present work so far build on deterministic formulations. Therefore we start with a short recapitulation of the simplest deterministic SIS model. Here the number of infected individuals *X*(*t*) is modeled as a smooth, deterministic function of time. The SIS model is then given by the ordinary differential equation

2 $$\begin{aligned} \frac{dX(t)}{dt}= & {} \beta X(t)\left( N-X(t)\right) -\gamma X(t),\nonumber \\ X(0)= & {} x_{0}\in (0,N]. \end{aligned}$$

In view of (2), Eq. (2) is sufficient to describe the whole system $\left( X(t),Y(t)\right)$. The properties of model (2) are well known, as it is equivalent to the logistic equation used to describe population growth in ecology, see e.g. Murray (1989). Because of its simplicity, Eq. (2) can be solved explicitly, which leads to

$$\begin{aligned} X(t)=\frac{N\beta -\gamma }{\beta -e^{-(N\beta -\gamma )\left( t-\frac{1}{x_{0}}\right) }} \end{aligned}$$

if $$\frac{N\beta }{\gamma }\ne 1$$, and

$$\begin{aligned} X(t)=\frac{1}{\beta t+\frac{1}{x_{0}}} \end{aligned}$$

if $\frac{N\beta }{\gamma }=1$. Clearly, *X*(*t*) remains within (0, *N*] if the process starts in this interval.

The asymptotic behaviour of the deterministic model (2) depends on the basic reproduction number, i.e. the expected number of secondarily infected individuals by a single initial case of infection,

3 $$\begin{aligned} R_{0}=N\beta /\gamma \end{aligned}$$

and can be characterized as follows: if $R_0\le 1$, the disease free equilibrium $X(t)=0$ is globally stable, while if $R_0>1$ the disease free equilibrium becomes unstable and the system has a unique (globally stable) endemic equilibrium

$$\begin{aligned} X^*=N-\frac{\gamma }{\beta }, \end{aligned}$$

fulfilling $\frac{dX(t)}{dt}=0$. Convergence here is monotonic with no oscillatory behaviour.

These facts provide the basis for answering the question, if and how it is possible to extinct the disease in the long run (asymptotically). Basically a decision maker may try to influence the paramters $\beta$ and $\gamma$ such that the condition for the condition for a disease free equilibrium is fulfilled.

### Diffusion-type SIS models

While many approaches for stochastic epidemiological models exist in literature, see e.g. Diekmann et al. (2013) or Chapter 6 of Keeling and Ross (2008), the present paper analyzes diffusion-type versions of the SIS model. Extending the simple deterministic SIS-model, this means that Eq. (2) is replaced by a stochastic (Ito) differential equation

4 $$\begin{aligned} dX(t)= & {} \left( \beta X(t)\left( N-X(t)\right) -\gamma X(t)\right) dt+\sigma (X(t))dW(t),\nonumber \\ X(0)= & {} x_{0}\in (0,N). \end{aligned}$$

Here, *W*(*t*) represents a (standard) Wiener process and the resulting stochastic process *X*(*t*) is adapted to the filtration generated by *W*(*t*). The measurable functions $\sigma (\cdot ):\,{\mathbb {R}}\rightarrow {\mathbb {R}}$ (the diffusion term) and $\beta (X(t))\left( N-X(t)\right) X(t)-\gamma X(t)$ have to fulfil some technical condition like a local Lipschitz condition together with a linear growth condition (see e.g. Fleming and Rishel 1975, p 118) or the Yamada–Watanabe condition (Yamada and Watanabe 1971), in order to guarantee existence and uniqueness of a (strong) solution.

The exact form of $\sigma$ depends on the viewpoint of modelling. Several types of diffusion terms $\sigma (\cdot )$ for SDE models have been proposed in epidemiologic literature. Keeling and Rohani (2008) distinguish four types of specifications. In the context of SIS models (4) we can rephrase their classification as follows:

1. *Constant noise*, $\sigma (\cdot )\equiv \sigma$ with $\sigma$ a positive real number. Such specifications are used often. As a result, in the long run the asymptotic expectation of the stochastic model equals the deterministic equilibrium. See e.g. Rohani et al. (2002) and McKane and Newman (2005). Unfortunately such an approach cannot exclude negative numbers of infected individuals.
2. *Scaled additive noise*, $\sigma (x)=\sqrt{\left( \beta \cdot x\cdot (1-x)+\gamma x\right) /N}$. For SIS models the analyzed stochastic differential equation (4) is used as a diffusion approximation (Kramers–Moyal approximation) of a continuous time (discrete state space) Markov chain with jump intensities $\beta$ and $\gamma$. Note that here *X*(*t*) models fractions of the population size *N*, which means that the drift term in (4) is replaced by $\beta \cdot x\cdot (1-x) - \gamma x$. Different approaches for diffusion approximation can be found e.g. in Gardiner (2009, Chapter 11). See also further applications to epidemiology in Fuchs (2013), Keeling and Grenfell (1999) and Bjornstad et al. (2002).
3. *External parameter noise*, $\sigma (x)=\sigma \cdot x(N-x)$. While cases (1) and (2) assume that the number of infected individuals is subject to random fluctuations, the basic assumption here is that the strength of infection of an—at first glance—deterministic epidemiological model is disturbed by random noise due to external, unpredictable forces. For possible underlying mechanisms see e.g. Abad et al. (1994), Albina (1997) and Dexter (2003).
4. *Heterogeneous parameter noise*, $\sigma (x)=\beta (x)\sqrt{x}(N-x)$. This approach also assumes noisy parameters. Different to case (3), The noise comes from fluctuations in individual behaviour. See Keeling and Rohani (2008).

In this paper we analyze two modifications of Eq. (4): The first variant develops further a recent discussion on stochastic SIS models with external parameter noise (case 3. above) and saturated incidence. Saturated incidence slightly generalizes the pure SIS-setup, but still one can argue that the resulting process fits into the SIS-framework. The second variant is the setup (4) with scaled additive noise, i.e. case 2. above. The main qualitative difference between these specification is the existence of a (nontrivial) stationary distribution (under some condition) in the first case and the absence of a stationary distribution in the second case.

Gray et al. (2011) analyze a stochastic SIS model with external parameter noise and use the specification $\sigma (X(t))=\sigma \cdot (N-X(t))X(t)$. Based on stochastic calculus, they prove necessary conditions for exponential extinction and on the other hand for the existence of a stationary solution. Chen and Kang (2014) generalize the setup by introducing saturated incidence to the stochastic model: the linear force of infection is replaced by the nonlinear term $\beta X/(1+h X)$, a specification introduced in a deterministic context by Capasso and Serio (1978) in order to model a decrease in the force of infection (e.g. by more cautious behaviour) if the number of infected individuals goes up. Chen et al. give necessary conditions for exponential extinction and for persistence in the mean. Furthermore, they prove the existence of a unique stationary distribution under certain conditions. Similar analysis of the slightly more complicated case of a stochastic SIRS model is given in Lahrouz et al. (2015). It also should be mentioned that in similar manner Lin et al. (2014) prove existence and uniqueness of a stationary solution for a SIS model with vaccination, which is more complex than the simple SIS model because of the additional class of vaccinated individuals.

While the purely probabilistic methods used by Gray et al. (2011) and Chen and Kang (2014) are interesting on their own, the present paper aims at a different approach and uses Fokker–Planck (or Kolmogorov) equations and their properties for analyzing the processes and their asymptotic behaviour. In particular, Fokker–Planck (or Kolmogorov) equations describe the (transition) density function of a Markov process *X*(*t*) at any time *t*. They also can be used to derive and analyze stationary distributions. Unfortunately, Gray et al. (2011) do not even mention the paper Roberts and Saha (1999), which derives the stationary distribution of a slight generalization of the Gray model directly from the Fokker–Planck equation and exhaustively analyzes the different cases of stationary density and extinction. Lin et al. (2014) mention the Fokker–Planck equation, but make no attempt to use it beyond the mere proof of existence of a stationary distribution.

We investigate the more general model with saturated incidence which was proposed in Chen and Kang (2014). Compared to Gray et al. (2011) and Chen and Kang (2014) the present paper simplifies the argumentation related to the existence and uniqueness of stationary solutions. Moreover, we calculate the stationary distributions in closed form. Compared to Roberts and Saha (1999) we analyze the more general model with saturated incidence and improve the argumentation on existence and uniqueness. In particular, the effects of boundaries and the possibility of additional weak solutions is taken into account. Finally, this paper not only analyzes stationary distributions but also treats exhaustively the limiting behaviour of the process, i.e. the weak convergence to one of the stationary distributions, depending on the parameter values.

The second case, a diffusion type SIS model with scaled additive noise, approximates a continuous time Markov chain model with discrete state space (number of infected), which in some sense can be considered as “the” basic SIS model. Important analysis on the discrete state space model has been done in e.g. Barbour (1976), Kryscio and Lefévre (1989) and the monograph Nasell (2011). There is no nontrivial stationary distribution in the discrete model, so questions about extinction times and quasi-stationary distributions move to the front. It turns out that SIS models with scaled additive noise inherit this property from the discrete model. In the present paper we therefore analyze basic properties of this process, in particular quasi-stationarity and the related extinction times for the diffusion approximation.

Finally, we analyze (optimal) decision making within the framework of this paper. The properties of stationary or quasi-stationary distributions can be used to influence the process parameters $\beta ,\gamma ,\sigma$ in a favorable (maybe even optimal) way. Our main results will answer the questions of how to set the parameter values in order to achieve extinction of the disease. In a separate section we also analyze a decision maker who is able to influence the level of treatment intensity (recovery rate $\gamma$) and who aims at minimizing the long term expected average costs for our first model and at minimizing the long term expected costs from the quasi stationary distribution for our second model.

### Structure

The remaining paper is structured as follows: In Sect. 2 the stochastic SIS model with saturated incidence it analyzed. In particular we give a deep analysis of the related stationary (limiting) distributions. In Sect. 3 we apply the Fokker–Planck approach to the Kramers–Moyal diffusion approximation of the original stochastic SIS model with discrete state space and analyze the related times to absorption and possible quasi stationary distributions. Section 4 develops the decision problem of choosing the optimal level of treatment intensity. The paper is concluded by Sect. 5.

## A stochastic SIS-model with external parameter noise and saturated incidence

The stochastic SIS model with saturated incidence by Chen and Kang (2014) can be formulated using the stochastic differential equation

5 $$\begin{aligned} dX(t) = \left( \beta \frac{X(t)\left( N-X(t)\right) }{1+hX(t)}-\gamma X(t)\right) dt+\sigma \frac{X(t)\left( N-X(t)\right) }{1+hX(t)}dW(t) \end{aligned}$$

Clearly the model in Gray et al. (2011) is a special case with $h=0$.

Compared to the deterministic model (2), the purely deterministic term $\beta dt$ in (2) is formally replaced by the stochastic term $\beta dt + \sigma dW(t)$. As discussed in the introduction (case 3. in the introduction), external parameter noise), this models fluctuations of the parameter $\beta$, i.e. uncertainty related to contagion, whereas the length of the recovery period is still assumed to be deterministic.

We assume that the process *X* starts within the interval (0, *N*). Equation (5) holds almost surely for any starting value and can be used to describe the process conditional on starting points $X(0)=x_{0}\in (0,N)$.

Because often the starting value *X*(0) cannot be observed in reality, throughout this paper we consider *X*(0) as a random variable with probability density $p_{0}(x)$. The case of an observable starting value still can be treated as the special case $p_{0}(x)=\delta (x-x_0)$ (the point mass at $x=x_0$), where $\delta$ denotes the Dirac delta function. In any case we assume that the process starts in the interval (0, *N*], which means that $p_0(\cdot )$ equals zero outside this region.

The nonlinear force of infection used in (5) is known as “saturated incidence” and was introduced into the epidemiological discussion by Capasso and Serio (1978). In this way it is possible to model a decline of the strength of infection $\beta /(1+h X(t))$ when the number of infected increases. Such an effect might be caused by more cautious behaviour and disease control, when the number of infected increases. For *X*(*t*) near zero the force of infection tends to $\beta$, whereas for *X*(*t*) near *N* the strength of infection tends to $\beta /(1+h N)$, which is smaller than $\beta$, given that $h>0$.

### Kolmogorov-forward equation

Denoting the density of any random variable *X*(*t*) (number of infected individuals at time *t*) by *p*(*x*, *t*), the related Kolmogorov forward, or Fokker–Planck equation (see e.g. Fleming and Rishel 1975, Theorem IX 8.1), which describes the evolution of the density *p*(*x*, *t*) over time, is given by

6 $$\begin{aligned} \frac{\partial p(x,t)}{\partial t}= & {} -\frac{\partial }{\partial x}\left[ \!\left( \beta \frac{x\left( N-x\right) }{1+hx}-\gamma x\right) p(x,t)\!\right] +\frac{1}{2}\sigma ^{2}\frac{\partial ^{2}}{\partial x^{2}}\left[ \!\frac{x^{2}\left( N-x\right) ^{2}}{\left( 1+hx\right) ^{2}}p(x,t)\!\right] \nonumber \\ p(x,0)= & {} p_{0}(x), \end{aligned}$$

where $p_0$ again denotes the (estimated) initial density of the process. If $X(0)=x_{0}$ is known with certainty, the initial condition reduces to $p_{0}(x)=\delta (x-x_{0})$.

**Fig. 1:**
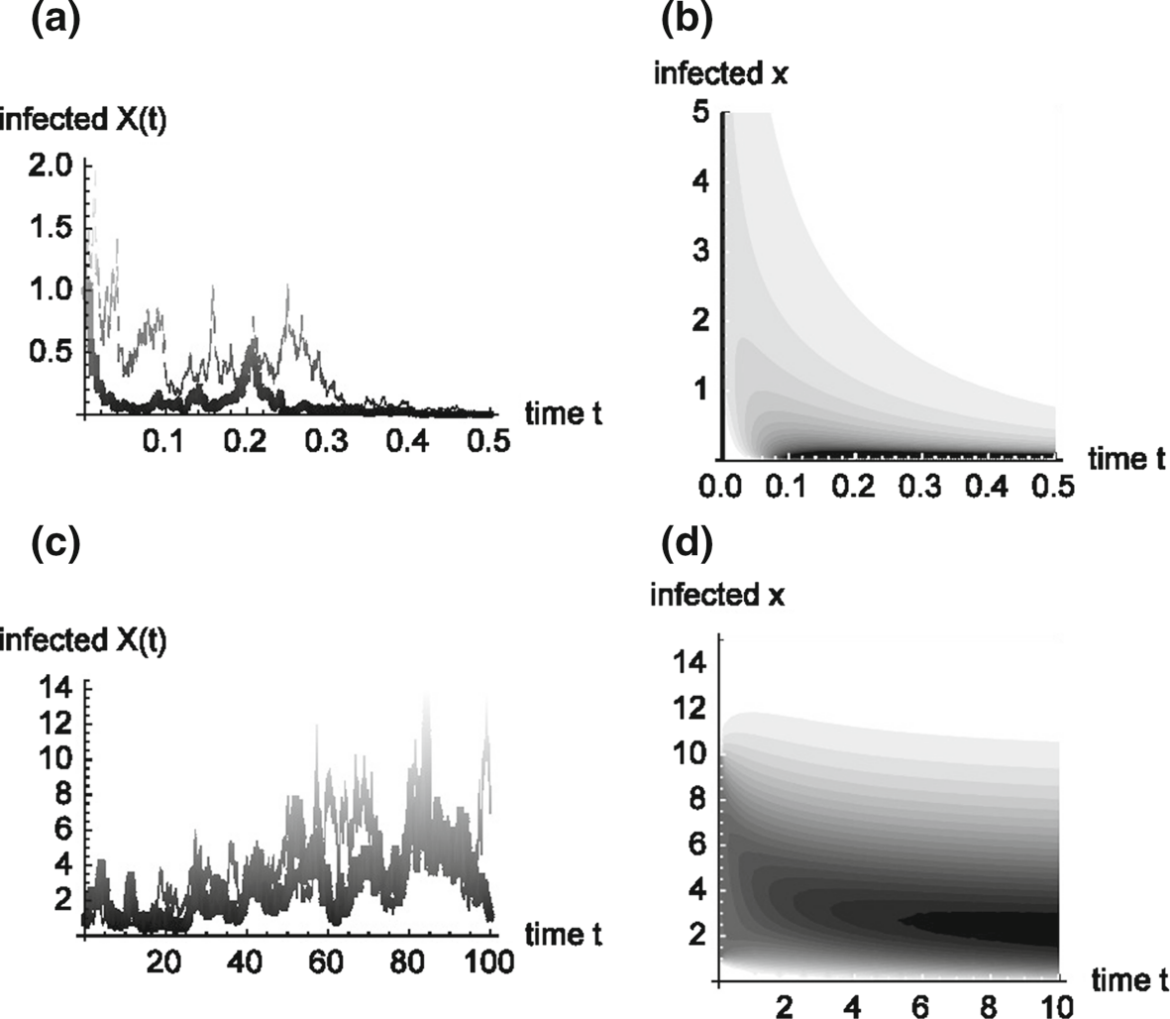
First line—Stochastic SIS model with saturated incidence with $N=100,\,\beta =0.013,\,\gamma =1,\,\sigma =0.06,h=0.05$—simulated paths (**a**) and contour plot (**b**) of the function *p*(*x*, *t*). Second line—Stochastic SIS model with saturated incidence with $N=100,\,\beta =0.013,\,\gamma =1,\,\sigma =0.005,h=0.05$—simulated paths and contour plot of the function *p*(*x*, *t*)

We use the notation

$$\begin{aligned} A(x)=\beta \frac{x\left( N-x\right) }{1+hx}-\gamma x \end{aligned}$$

and

$$\begin{aligned} B(x)=\sigma ^{2}\frac{x^{2}\left( N-x\right) ^{2}}{\left( 1+hx\right) ^{2}}. \end{aligned}$$

These functions are continuous (therefore also measurable) and bounded on the interval [0, *N*].

The diffusion term *B*(*x*) equals zero at two points, $x=0$ and $x=N$. Because the process starts within the interval (0, *N*), we have to consider the related boundary conditions. At both boundaries the derivative of *B* equals zero, so there are prescribed boundaries: at $x=N$ the drift is negative, $A(N)=-\gamma N$, hence there is an entrance boundary which is never reached. On the other hand, at $x=0$ we have $A(x)=0$, which indicates a natural boundary (see e.g. Gardiner 2009, p. 119). In consequence, $x=0$ is absorbing but is also never reached. Altogether, by continuity of the sample paths, any process *X*(*t*) starting in (0, *N*) will stay within this interval forever. Consequently, because on [0, *N*] all parameter functions of the process are Lipschitz continuous and bounded, there exists a unique strong solution of SDE (5) on this interval (see e.g. Fleming and Rishel 1975, Theorem V 4.1).

In order to solve the Fokker–Planck equation (6), one has to account for the nature of the boundary points. In particular, the forward Fokker–Planck equation with prescribed boundary conditions requires the additional condition

7 $$\begin{aligned} A(N)p(N,t)-\frac{1}{2}\frac{\partial }{\partial x}\left[ B(N)p(N,t)\right] =0, \end{aligned}$$

(reflecting boundary) at $x=N$ in order to have a unique solution. At zero, no boundary condition is needed [see Feller (1952) and e.g. the synopsis in Appendix A of Cacio et al. (2012)].

Figure 1 shows two instances of the process *X*(*t*) and the related density function *p*(*x*, *t*) over time. Parts (a), (c) respectively show two simulated trajectories of the respective processes, while (b), (d) are contour plots of the time dependent density function *p*(*x*, *t*). The contour lines depict combinations of *x* and *t* with equal density *p*(*x*, *t*). Regions with higher density are depicted in darker nuances. The density at time zero is assumed as a uniform distribution on $[1,\,10]$.

While the case $N=100,\,\beta =0.013,\,\gamma =1,\,\sigma =0.06,h=0.05$ shows fast extinction of the disease, reducing the volatility to $\sigma =0.005$ seemingly leads to endemic behaviour of the system. We will see in the following that these conjectures are indeed true.

### Stationary distribution

Stationary distributions can be analyzed with the help of the Fokker–Planck equation. If $p(x)=p(x,t)$ is the density of the stationary distribution, we must have $\frac{dp(x,t)}{dt}=0$. Plugging this into the Fokker–Planck equation (6), we see that the stationary distribution is a solution of the equation

$$\begin{aligned} \frac{\partial }{\partial x}\left[ A(x)p_{s}(x)\right] =\frac{1}{2}\frac{\partial ^{2}}{\partial x^{2}}\left[ B(x)p_{s}(x)\right] . \end{aligned}$$

This means that the probability flux

$$\begin{aligned} J(x,t)=A(x)p(x,t)-\frac{1}{2}\frac{\partial }{\partial x}\left[ B(x)p(x,t)\right] \end{aligned}$$

must be constant over *x*, if $p(x,t)=p_{s}(x)$. Because the boundaries at *N* and zero are reflecting, respectively natural, no probability mass is lost at any time (i.e. $J(x,t)=0$), so the stationary density $p_{s}(\cdot )$ fulfills

8 $$\begin{aligned} A(x)p_{s}(x)-\frac{1}{2}\frac{\partial }{\partial x}\left[ B(x)p_{s}(x)\right] =0. \end{aligned}$$

#### Fact 1

Classical, i.e. $C^2((0,N))$, solutions of (8) have the form

9 $$\begin{aligned} p^C_{s}(x)=C\cdot (1+hx)^{2}e^{-\frac{2\gamma (1+hN)^{2}}{N\sigma ^{2}(N-x)}}(N-x)^{2\cdot \left( 1-\frac{(hN+1)(N(\beta +h\gamma )-\gamma )}{N^{2}\sigma ^{2}}\right) }x^{2\cdot \left( \frac{N\beta -\gamma }{N^{2}\sigma ^{2}}-1\right) } \end{aligned}$$

with some $C>0$. Moreover the equation has also a nontrivial solution on [0,N], namely the weak solution $p_W(x)=\delta (x)$, where $\delta$ denotes the Dirac delta function. This is the point mass at zero (“extinction”).

Finally, all linear combinations of $p_s^C$ and $\delta$ are solutions of (8).

#### Proof

The $C^2$ solution can be checked by plugging (9) into the equation. In order to see that $\delta (x)$ is a weak solution, note that

$$\begin{aligned} \int _{0}^{N}A(x)\delta (x)\varphi (x)dx-\frac{1}{2}\int _{0}^{N}B(x)\delta (x)\frac{\partial \varphi (x)}{\partial x}dx=A(0)\varphi (0)-\frac{1}{2}B(0)\frac{\partial \varphi (0)}{\partial x}=0 \end{aligned}$$

for any test function $\varphi$ with compact support on $\left( 0,N\right)$. Because *J* acts as a linear operator on *p*, all linear combinations of $p_s^C$ and $\delta$ are solutions of (8). $\square$

The Dirac delta function may be interpreted as a generalized density of a random variable concentrated at $x=0$ with probability one and therefore qualifies as a stationary density on [0, *N*]. For the $C^2$ solutions (9) the situation is more complicated. Choosing a positive constant *C* ensures nonnegativity of $p^C_s(x)$. In order to ensure that $p^C_{s}(\cdot )$ is a density, *C* has to be chosen such that

$$\begin{aligned} \int _{0}^{N}p_{s}^{C}(x)dx=1. \end{aligned}$$

If this is possible, we define $p_s(x)=p^C_s(x)$. In this case it makes sense to extend the density to the whole set ${\mathbb {R}}$ by extending it as zero outside *D*. Note that because of $\lim _{x\uparrow N}p_s(x)=0$, the extension is continuous at $x=N$. This also ensures that boundary condition (7) is fulfilled.

These facts imply that either (if $p_s$ is not defined) the set ${\mathcal {I}}$ of stationary densities is given by ${\delta }$ or (if $p_s$ is defined) by the set of convex combinations of $\delta$ and $p_s$. Moreover, $\delta$ and $p_s$ (if defined) are extremals of the set ${\mathcal {I}}$ (i.e., they can not be written as combinations of other invariant measures) and hence the related probability measures are ergodic invariant measures^2^, compare Kallenberg (2002, Theorem 10.26).

In the following we will characterize the cases when $p_s$ exists and analyze the question of weak convergence to a stationary distribution.

### Asymptotic behavior: convergence to stationary densities and extinction

We can now state a condition for the existence of a nontrivial stationary density, i.e. an “endemic equilibrium”.

#### Proposition 1

$p^C_s(x)$ in (9) is integrable (i.e. there exists a *C* such that $p_s(x)=p^C_s(x)$) is a density) if and only if the modified basic reproduction number $R_1$ fulfills^3^

10 $$\begin{aligned} R_1:=R_{0}-\frac{1}{2}\frac{N^{2}\sigma ^{2}}{\gamma }>1, \end{aligned}$$

with $R_{0}$ as defined in (3).

#### Proof

This can be seen from the fact that (10) is equivalent to $m=2\left( 1-\frac{N\beta -\gamma }{N^{2}\sigma ^{2}}\right) <1$, which implies finiteness of the integral $\int _{0}^{N}\frac{1}{x^{m}}dx$ and therefore—by the limit comparison test—finiteness of $\int _{0}^{N}p^C_{s}(x)dx$. With $C=\left( \int _{0}^{N}p^C_{s}(x)dx\right) ^{-1}$ we get that $p_s(x)$ is a density. $\square$

Observe that for a fixed $\sigma ^2$ the basic reproduction number $R_0$ (which does not depend on $\sigma ^2$) must be higher in order to achieve a stationary density than it has to be in the deterministic model to achieve an endemic equilibrium. Also note that the critical inequality (10) does not depend on the saturation parameter *h*. Hence models with ($h>0$) and without saturation ($h=0$) show qualitatively the same asymptotic behaviour, although the exact form of the stationary density (9) depends on the saturation parameter *h*.

If (10) holds, the stationary density $p_s(x)$ converges to zero when *x* goes to *N* as pointed out above. However, varying behaviour is possible at $x=0$, depending on the exact values of the parameters.

#### Proposition 2

If

11 $$\begin{aligned} R_2:=R_{0}-\frac{N^{2}\sigma ^{2}}{\gamma }\ge 1, \end{aligned}$$

then there is a stationary density $p_s(x)$ and we have

$$\begin{aligned} \lim _{x\rightarrow 0^{+}}p_{s}(x)={\left\{ \begin{array}{ll} 0 &{}\quad \text {if }R_{0}-\frac{N^{2}\sigma ^{2}}{\gamma }>1\\ C\cdot e^{-\frac{2(1+hN)^{2}}{R-1}}N^{2\cdot \left( 1-\frac{(hN+1)(N(\beta +h\gamma )-\gamma )}{N^{2}\sigma ^{2}}\right) } &{}\quad \text {if }R_{0}-\frac{N^{2}\sigma ^{2}}{\gamma }=1. \end{array}\right. } \end{aligned}$$

#### Proof

Condition (11) implies (10), the condition for a density $p_s(x)$. With $D=\frac{\beta N-\gamma }{N^{2}\sigma ^{2}}-1\ge 0$ that $x^{2D}=1$ for $x>0$ and $D=0$, and $\lim _{x->0^+}x^{2D}=0$ if $D>0$. This gives the two cases in (12). $\square$

In the first case the (extended) density is continuous at zero, in the second case it converges to a finite value from the right. If (11) is violated, we have $\lim _{x\rightarrow 0}p_s(x)=+\infty$ but $p_s(x)$ is still integrable.

Finally, if (10) is violated, no classical solution exists for the flux-equation (8), because then (9) is not integrable between zero and *N*. Still there is the weak solution $\delta (x)$, which represents the discrete distribution concentrated almost surely at $x=0$, i.e. complete extinction of the disease.

While we have identified the possible stationary solutions, the question remains whether the densities *p*(*x*, *s*) converge to one of the densities $p_s(x)$ or $\delta (x)$ if *t* goes to infinity. First, observe that the considered model fulfills the conditions of Theorem 2.1 in Zhang and Chen (2013) on the open interval $D=(0,N)$. In particular *D* is an irreducible set of recurrent states. Therefore there exists a unique limiting stationary distribution.

This is true for any parameter constellation. However, a decision maker would like to know more. In particular it would be important to know whether there are parameter constellations that lead to extinction of the disease (which is a stationary case as shown above) in the long run.

The following theorem gives the answer that the densities $p(\cdot ,t)$ converge (depending on the exact parameter values) to one of the two ergodic invariant cases $\delta$ (extinction) or $p_s$ (endemic distribution). Moreover, in order to achieve asymptotic extinction of the disease, the transmission coefficient $\beta$, the recovery rate $\gamma$ and the volatility parameter $\sigma$ have to be set such that the modified basic reproduction number $R_1$—see (10)—becomes smaller or equal to one. This holds independently of the starting distribution. In practice this can be achieved by preventive activities ($\beta$), e.g. education and vaccination, and by influencing the recovery process ($\gamma$), e.g. by increasing the efficiency of treatment. Usually it will not be easy to influence the uncertainty in parameter $\beta$, modelled by the volatility parameter $\sigma$. Finally, the saturation parameter *h* does not play a role in the basic criterion for extinction.

#### Theorem 1

If the relation

12 $$\begin{aligned} R_1>1, \end{aligned}$$

(the condition of Proposition 1) holds, the density *p*(*x*, *t*) converges to the stationary density $p_s(x)$ as *t* goes to infinity,

$$\begin{aligned} (A)\;\lim _{t\rightarrow \infty }p(x,t)=p_{s}(x). \end{aligned}$$

If (12) is violated, then

$$\begin{aligned} (B)\;\lim _{t\rightarrow \infty }p(x,t)=\delta (x). \end{aligned}$$

#### Proof

If (10) holds then the classical solution $p_s(x)$ exists. Moreover, any classical solution of the Fokker Planck equation (6)—in fact the density *p*(*x*, *t*)—is defined on (0, *N*) and can be written (see e.g. Gardiner 2009, 5.4.1 and 5.4.2) as

13 $$\begin{aligned} p(x,t)=\sum _{i=0}^{\infty }H_{i}\bar{p}_{i}(x)e^{-\lambda _{i}t}, \end{aligned}$$

where the $\lambda _{i}$ and $\bar{p}_{i}(z)$ are the eigenvalues and the related eigenfunctions of the eigenvalue equations

14 $$\begin{aligned} -\frac{\partial }{\partial x}\left[ A(x)p_{i}(x)\right] +\frac{1}{2}\frac{\partial ^{2}}{\partial x^{2}}\left[ B(x)p_{i}(x)\right] =-\lambda _{i}p_{i}(x). \end{aligned}$$

The coefficients $H_{i}$ are given by

$$\begin{aligned} H_{i}=\int _{0}^{N}\bar{q}_{i}(x)p_{0}(x)\,dx \end{aligned}$$

and the functions $\bar{q_{i}}$ are solutions of the dual eigenvalue equations

15 $$\begin{aligned} A(x)\frac{\partial }{\partial x}q_{i}(x)+\frac{1}{2}B(x)\frac{\partial ^{2}}{\partial x^{2}}q_{i}(x)=-\lambda _{i}q_{i}(x). \end{aligned}$$

Note that (see e.g. Risken 1989) the sets of eigenvalues coincide for (14) and (15). The eigenvalues are nonnegative real and can be assumed to be sorted in ascending order in the following. The stationary distribution $p_{s}(x)$ is the (normalized) eigenfunction $\bar{p}_{0}$ related to the eigenvalue $\lambda _{0}=0$. Moreover, $\bar{q}_{0}=1$ and therefore $H_{0}=1$, see e.g. Gardiner (2009, p. 125).

**Fig. 2:**
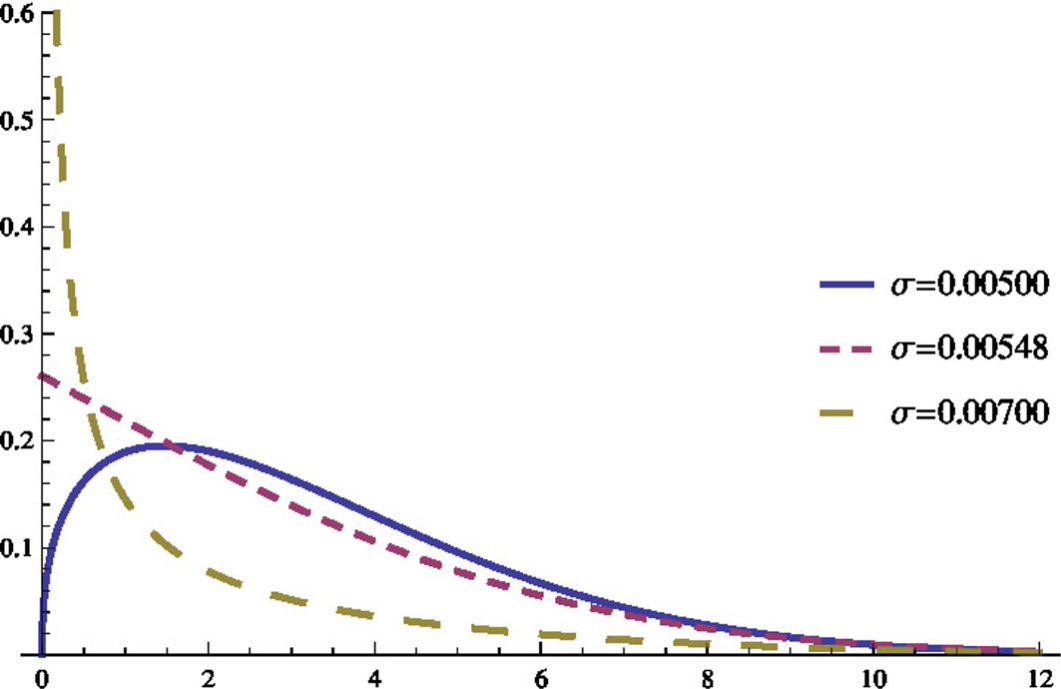
Three stationary densities (number of infected at the horizontal axis) with $N=100,\,\beta =0.013,\,\gamma =1,\,h=0.05$, but different volatility parameter $\sigma$

Putting these facts together, it can be seen that

$$\begin{aligned} \lim _{t\rightarrow \infty }p(z,t)=p_{s}(x)+\lim _{t\rightarrow \infty }\sum _{i=1}^{\infty }H_{i}\bar{p}_{i}(x)e^{-\lambda _{i}t}=p_{s}(x). \end{aligned}$$

If (10) is violated, then (13) still holds, but there is no eigenvalue zero. This means that $\lim _{t\rightarrow \infty }p(z,t)=0$ for any $x\in (0,N)$: there is no stationary distribution on (0, *N*). The probability mass therefore must be concentrated in the points $x=0$ and $x=N$. However, it is not possible that in the limit there is a positive probability at $x=N$, because this is an entrance boundary. Therefore, in the limit the whole probability mass is concentrated at $x=0$, i.e. the delta function $\delta (x)$ then remains as the only limiting stationary (generalized) density on [0, *N*]. $\square$

#### Remark 1

Because of (13), convergence to the disease free equilibrium is exponential with rate $\lambda _1>0$, the smalles positive eigenvalue of (14).

Altogether we can distinguish four cases: If (11) holds, a nontrivial stationary density exists. If *x* goes to zero this density either converges to zero, goes to a positive finite value, or to infinity. Which case is relevant, depends on the two cases in (12). If (11) is not fulfilled, but still (10) holds, a nontrivial stationary density exists, but it is unbounded at $x=0$. Finally, if (10) is violated, no normalizing constant exists, (9) is not related to a stationary density and the stationary distribution is given by the disease free equilibrium.

Figure 2 shows the three cases of (classic) stationary density functions by example. The parameters $N,\beta ,\gamma ,h$ are fixed at the same values as before. The first case, $\sigma =0.005$, in fact is the second case of Fig. 1 and fulfills (11), so we actually have a stationary density for this example. The second case, $\sigma \approx 0.00548$, which still leads to integrability, fulfills (11) with equality, see also the second case in (12). The final case, $\sigma =0.007$ violates (11) but still fulfills the critical integrability condition (10). Clearly the first case of Fig. 1 violates the critical condition, hence no normalizable density exists.

The behavior of the density $p_s(x)$ at $x=0$ can be analyzed further. To this end, observe

16 $$\begin{aligned} \frac{\partial p_{s}(x)}{\partial x}= -\frac{2e^{-\frac{2\gamma }{N\sigma ^{2}(N-x)}}(N-x)^{\frac{2\gamma -2\beta N}{N^{2}\sigma ^{2}}-4}x^{\frac{2\beta N-2\gamma }{N^{2}\sigma ^{2}}-3}\left( \gamma +(N-x)\left( \sigma ^{2}(N-2x)-\beta \right) \right) }{\sigma ^{2}}. \end{aligned}$$

Consider now the limit $x\downarrow 0$. The last factor in (16) stays negative in the case (A) of (12), it is positive if (11) is violated (but (10) still holds) and equals zero in the case (B) of (12). Moreover, $x^{\frac{2\beta N-2\gamma }{N^{2}\sigma ^{2}}-3}$ goes to zero if $R_{0}-\frac{3}{2}\frac{N^{2}\sigma ^{2}}{\gamma }>1$, goes to infinity if $R_{0}-\frac{3}{2}\frac{N^{2}\sigma ^{2}}{\gamma }<1$ and goes to one if $R_{0}-\frac{3}{2}\frac{N^{2}\sigma ^{2}}{\gamma }=1$. The other factors in (16) converge to positive (finite) numbers. Together this means that in the first case of (12) the slope of $p_{s}$ at zero is zero, $+\infty$ or a finite positive value, depending on the value of $R_{0}-\frac{3}{2}\frac{N^{2}\sigma ^{2}}{\gamma }$. If a density $p_s(x)$ exists but (11) is violated, then the slope is $-\infty$ and in in the second case of (12) the slope is zero. Note also that in view of (16) (and depending on the concrete values of the parameters $\beta ,\,\gamma$ and $\sigma$) the density $p_{s}(x)$ may possess up to two peaks.

## A stochastic model with scaled additive noise

Consider now the stochastic differential equation

17 $$\begin{aligned} dZ(t)= & {} \left[ \beta Z(t)\left( 1-Z(t)\right) -\gamma Z(t)\right] dt+\frac{\sqrt{\beta Z(t)\left( 1-Z(t)\right) +\gamma Z(t)}}{\sqrt{N}}\, dW(t)\nonumber \\ Z(0)= & {} x_{0}\in (0,N], \end{aligned}$$

with the related Fokker–Planck equation

18 $$\begin{aligned} \frac{\partial p(z,t)}{\partial t}= & {} -\frac{\partial }{\partial z}\left[ \left( \beta z\left( 1-z\right) -\gamma z\right) p(z,t)\right] +\frac{1}{2N}\frac{\partial ^{2}}{\partial z^{2}}\left[ \left( \beta z\left( 1-z\right) +\gamma z\right) p(z,t)\right] \nonumber \\ p(z,0)= & {} p_{0}(z). \end{aligned}$$

This model can be used to describe the dynamics of the fraction $Z(t)=\frac{X(t)}{N}$ of infected individuals in a population of size *N*.

This is a model with scaled additive noise (case 2. in the classification at page 4). While the stochastic model in the previous section can be derived from a deterministic model by introducing randomness in one of the parameters, (17) does not need such external effects and can be derived from a genuinely stochastic model with the same parameters $\beta ,\,\gamma$. In fact, consider a continuous time Markov chain, where the number of infected individuals *X*(*t*) takes values in $\left\{ 0,1,\ldots ,N\right\}$, the process *X*(*t*) has right continuous sample paths and a probability measure *P* is related such that

19 $$\begin{aligned} P\left[ X_{t+h}=x_{t}+1\,|\,X_{t}=x_{t}\right]= & {} \frac{\beta }{N} x_{t}\left( N-x_{t}\right) h+o(h),\nonumber \\ P\left[ X_{t+h}=x_{t}-1\,|\,X_{t}=x_{t}\right]= & {} \gamma x_{t}h+o(h)\nonumber \\ P\left[ X_{t+h}=x_{t}\,|\,X_{t}=x_{t}\right]= & {} 1-\frac{\beta }{N} x_{t}\left( N-x_{t}\right) h-\gamma x_{t}h+o(h) \end{aligned}$$

and $P\left[ X_{t+h}=x_{t}+k\,|\,X_{t}=x_{t}\right] =o(h)$ in all other cases $k\notin \{0,1,-1\}$.

Starting with (19) and going to fractions *Z* instead of absolute numbers *X*, the Fokker–Planck equation (18) can be derived as the Kramers–Moyal expansion of the Master equation related to the Markov chain (19). Finally is the SDE related to the Fokker–Planck equation. See e.g. section 11.2 of Gardiner (2009) for the general approach, or section 4.3 in Fuchs (2013), which also includes further examples from epidemiology.

It should be noted that the continuous state space model (17) as an expansion of the discrete state space model (19) works well only if the volatility parameter $\sigma$ is not too large. See e.g. Ovaskainen and Meerson (2010) on the Kramers–Moyal approach and alternative expansions in ecological modelling.

In the following we use the notation

$$\begin{aligned} A(z)=\beta z\left( 1-z\right) -\gamma z \end{aligned}$$

and

$$\begin{aligned} B(z)=\frac{\beta z\left( 1-z\right) +\gamma z}{N}. \end{aligned}$$

Looking at the drift term of (17), this is definitely a SIS model. However, now the process *Z*(*t*) denotes the proportion of infected individuals in a population of size *N*, i.e. $Z(t)=X(t)/N$. We have $B(z)=0$ at $z=0$ and at $z=1+\gamma /\beta$. If $p_{0}(\cdot )$ has its support in $[0,1+\gamma /\beta ]$, then the process will stay in this interval at any time $t\ge 0$. Using the Yamada–Watanabe condition (Yamada and Watanabe (1971)) it is possible to show that because $A(\cdot )$ is Lipschitz continuous and $\sqrt{B(\cdot )}$ is Hölder continuous on the interval $[0,1+\gamma /\beta ]$, solutions of (17) are pathwise unique (see e.g. Altay and Schmock 2013, Corollary 2.19).

In order to use the Fokker–Planck equation (18) one has to combine it with the boundary conditions $p(0,t)=0$ (an absorbing state at $x=0$). The second boundary condition $J(1+\gamma /\beta )=0$ (a reflecting boundary at $1+\gamma /\beta$) is always true for (20) and (20).

While the extension of the state space to the larger interval $[0,1+\gamma /\beta ]$ is an unpleasant fact, it can be seen easily that for $x>1-\gamma /\beta$—which would be the deterministic (i.e. for $\sigma =0$) endemic equilibrium—the drift term *A*(*z*) is negative and its magnitude increases with *z*, whereas the diffusion term $\sqrt{B(z)}$ decreases to zero, when *z* approaches $1+\gamma /\beta$.

### A nontrivial stationary distribution does not exist

Note that the Markov chain (19) has a finite state space and all states—with the exception of zero—are transient. Zero is the only absorbing state and it is reachable from the other states. As a result, the stationary distribution of the Markov chain is trivial, because irrespectively of the starting point the process finally is absorbed at zero. It is possible to be absorbed at zero in finite time, which is a main distinction from the stochastic model with saturated incidence (5) and also the simple deterministic model (2). As we will see, the approximating stochastic model (17), (18) keeps this important property of the Markov chain (19), which is not true for some other approximation methods like e.g. the Van Kampen system size expansion.

There exists no stationary density on the interval $(0,1+\gamma /\beta )$. In fact, using (20), (20), the classical solution of the flux equation (8) is given by

20 $$\begin{aligned} p_{s}(z)=C\cdot \frac{Ne^{2Nz}(\beta +\gamma -\beta z)^{\frac{4N\gamma }{\beta }-1}}{z}. \end{aligned}$$

However, (20) is not integrable on $\left( 0,1+\gamma /\beta \right)$ because of the factor 1 / *z* and hence *C* cannot be chosen to normalize $p_{s}(z)$ as a density. On the other hand the flux equation still has the weak solution $p_{s}(z)=\delta (z)$, as (10) also holds when $A(\cdot ),\,B(\cdot )$ are defined by (20) and (20).

Again, $\delta (x)$ fulfills the Fokker–Planck equation and is therefore the only (generalized) stationary density of (17).

### Quasi-stationary distributions and the Yaglom-Limit

In the following we denote by $G(t)=\int _{0}^{1+\gamma /\beta }p(z,t)\,dz$ for $t>0$ the probability of being not absorbed at the disease free state $x=0$ up to time *t*, i.e. the probability that $X(t)>0$. The probability distribution of *Z*(*t*) can be described by the generalized density

$$\begin{aligned} P(z,t)=p(z,t)+(1-G(t))\delta (z). \end{aligned}$$

**Fig. 3:**
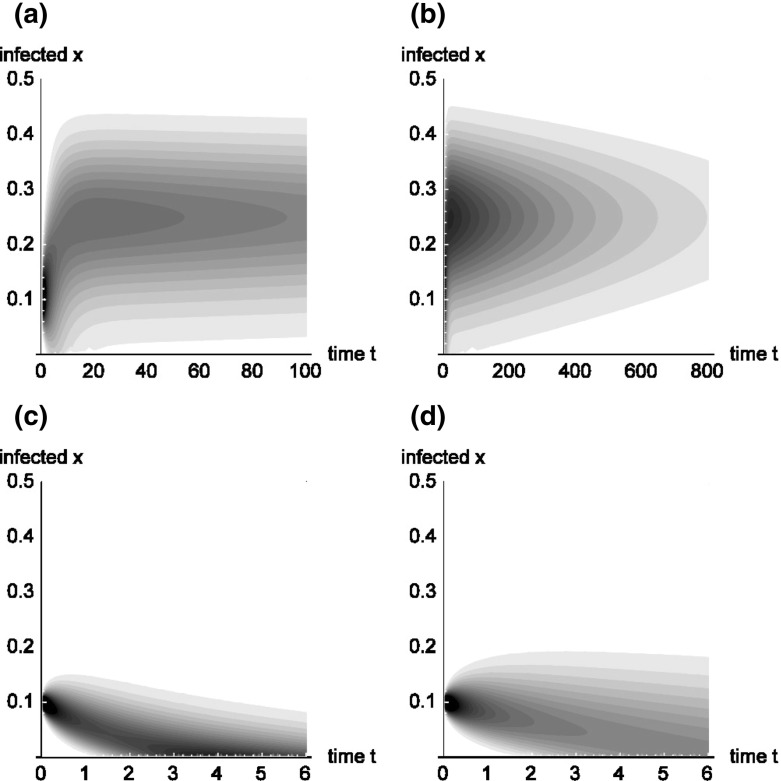
Contour plots of the function *p*(*x*, *t*) for different models. First line: Markov chain approximation with $\beta =1.1,\,\gamma =0.8$ and $N=100$. Plot (**a**) seems to show the building-up of a stationary distribution. Over a longer time horizon, (**b**) demonstrates that probability mass is lost over time, and there is no stationary distribution. In the second line (**c**) depicts the development for the model with $\beta =0.8,\,\gamma =1.1$ and $N=100$, and (**d**) shows the model $\beta =\gamma =0.9,\,N=100$. Both cases show quick extinction of the disease

Figure 3 shows three examples for the development of *p*(*x*, *t*) over time. In the first case, (a) and (b), extinction of the disease happens very slowly, whereas the cases (c) and (d) show quick extinction.

The qualitative differences in long-term behaviour can be analyzed further. An important question is the existence of a quasi-stationary density $\bar{p}(\cdot ):(0,1+\gamma /\beta )\rightarrow {\mathbb {R}}^+_0$, i.e. a time independent density for the conditional distribution of *Z*(*t*) given that extinction has not occurred at time *t*, i.e.

21 $$\begin{aligned} \bar{p}(z)=\frac{p(z,t)}{G(t)}. \end{aligned}$$

Closely linked is the question, whether such a quasi-stationary distribution can be obtained as a Yaglom-limit, i.e. that regardless of the start distribution $p_0$ we have

22 $$\begin{aligned} \bar{p}(z)=\lim _{t\rightarrow \infty } \frac{p(z,t)}{G(t)}. \end{aligned}$$

See e.g. Meleard (2012) and Collet et al. (2012) for these and further related notions.

Using (21), we substitute $p(x,t)=\bar{p}(x) G(t)$ into the Fokker–Planck equation (18) and get (after dividing by *G*(*t*))

23 $$\begin{aligned} \bar{p}(z) \frac{\partial G(t)}{G(t)\partial t} = -\frac{\partial }{\partial z}\left[ \left( \beta z\left( 1-z\right) -\gamma z\right) \bar{p}(z)\right] +\frac{1}{2N}\frac{\partial ^{2}}{\partial z^{2}}\left[ \left( \beta z\left( 1-z\right) +\gamma z\right) \bar{p}(z)\right] \end{aligned}$$

for potential quasi-stationary distributions.

Clearly the trivial solution $\bar{p}(z)=\delta (z)$ is a solution of (23). Nontrivial solution only may exist for constant

24 $$\begin{aligned} \frac{\partial G(t)}{G(t)\partial t}=:-\lambda . \end{aligned}$$

This means that nontrivial quasi-stationary densities must be eigenfunctions of the operator ${\mathcal {A}}$ defined by

$$\begin{aligned} {\mathcal {A}} f (z) = -\frac{\partial }{\partial z}\left[ \left( \beta z\left( 1-z\right) -\gamma z\right) \bar{f}(z)\right] +\frac{1}{2N}\frac{\partial ^{2}}{\partial z^{2}}\left[ \left( \beta z\left( 1-z\right) +\gamma z\right) \bar{f}(z)\right] , \end{aligned}$$

i.e. they fulfil the eigenvalue equation

25 $$\begin{aligned} -\lambda \, \bar{p}(z) = -\frac{\partial }{\partial z}\left[ \left( \beta z\left( 1-z\right) -\gamma z\right) \bar{p}(z)\right] +\frac{1}{2N}\frac{\partial ^{2}}{\partial z^{2}}\left[ \left( \beta z\left( 1-z\right) +\gamma z\right) \bar{p}(z)\right] \end{aligned}$$

with boundary condition $\bar{p}(0)=0$ to ensure absorption at zero and $\bar{p}(1+\gamma /\beta )=0$ which follows from the zero-flux condition, ensuring a reflecting boundary at $1+\gamma /\beta$.

Therefore, any nonnegative eigenfunction $\bar{p}_i$ of ${\mathcal {A}}$ with related (real) eigenvalue $\lambda _i$ that can be normalized is a candidate for a quasi-stationary density. Moreover, from (24) we see that the only survival probability $G_i$ consistent with $\lambda _i$ and $\bar{p}_i$ is given by

26 $$\begin{aligned} G_i(t)=e^{-\lambda _i\,t}. \end{aligned}$$

### The Yaglom-limit

The question remains, what happens, when *t* goes to infinity. Recall that the eigenvalues $\lambda _i$ are nonnegative (see e.g. Risken 1989) and in fact positive: there is no eigenvalue $\lambda =0$ because the related eigenfunction is (20), which can not be normalized. In the following we consider the eigenvalues $\lambda _i$ as ordered, such that $\lambda _1$ is the smallest eigenvalue. The following proposition describes the asymptotic behavior of the process.

#### Proposition 3

Consider the SIS model (18). The density function *p*(*x*, *t*) converges to the Dirac delta function, i.e.

27 $$\begin{aligned} \lim _{t\rightarrow \infty } p(x,t)=\delta (x). \end{aligned}$$

If the—normalized—eigenfunction $\bar{p}_1$ is a density then it is the Yaglom-limit of the process *Z*(*t*), i.e. (22) holds. Moreover, $\lambda _1$ is the rate at which the survival probability $G(t)=e^{-\lambda _1 t}$ decreases exponentially.

#### Proof

Define a function *q* such that

28 $$\begin{aligned} p(z,t)=\bar{p}_1(z) q(z,t), \end{aligned}$$

which (after substitution into the Fokker–Planck equation (18)) satisfies the backward equation

$$\begin{aligned} \frac{\partial q(z,t)}{\partial t} = A(z) \frac{\partial }{\partial z}q(z,t)+\frac{1}{2N}B(z)\frac{\partial ^{2}}{\partial z^{2}} q(z,t). \end{aligned}$$

We are interested in solutions of the form

29 $$\begin{aligned} q(z,t)=\bar{q}_i(z) e^{-\lambda _i}, \end{aligned}$$

which satisfy the eigenvalue equation

30 $$\begin{aligned} -\lambda _i \bar{q}_i= & {} A(z) \frac{\partial }{\partial z}\bar{q}_i(z)+\frac{1}{2N}B(z)\frac{\partial ^{2}}{\partial z^{2}} \bar{q}_i(z). \end{aligned}$$

Using (21), (26), (28) and (29) we get

31 $$\begin{aligned} \bar{p}_i(z)=\bar{p}_1(z)\bar{q}_i(z). \end{aligned}$$

Based on (25) and (30) it can be shown that $\bar{p}$ and $\bar{q}$ form a bi-orthogonal system, in particular

32 $$\begin{aligned} \int _0^{1+\gamma /\beta } \bar{p}_i(z)\bar{q}_j(z)=\delta _{ij}. \end{aligned}$$

Using this fact, we can write any solution of the Fokker–Planck equation (18) as a linear combination of the eigenfunctions, i.e.

33 $$\begin{aligned} p(z,t)=\bar{p}_1(z)e^{-\lambda _1 t}+\sum _{i=2}^\infty H_i \bar{p}_i(z)e^{-\lambda _i t}, \end{aligned}$$

where the coefficients $H_i$ depend on the initial condition in the following way:

34 $$\begin{aligned} A_i=\int _0^{1+\gamma /\beta }\bar{q}_i(z)p_1(z)\,dz. \end{aligned}$$

Note that because of (31) for $i=1$ we have $\bar{q}_i(z)=1$ and (34) reduces to $H_1=1$. From (33) it can be seen that the smallest eigenvalue allows to estimate the speed of convergence to the disease free state.

Recall now that the eigenvalues are ranked and positive. From (33) we can conclude $\lim _{t\rightarrow \infty }p(x,t)=0$ for $z\in (0,1)$. Again, $x=0$ is the only point left, where the probability mass is concentrated in the limit, i.e. (27) must hold.

Moreover, because of

$$\begin{aligned} \lim _{t\rightarrow \infty } \frac{p(z,t)}{e^{-\lambda _1}}=\lim _{t\rightarrow \infty }\hat{p}_1(z) + \sum _{i=2}^\infty A_i \hat{p}_i(z)e^{(-\lambda _i + \lambda _1) t}=\hat{p}_1(z), \end{aligned}$$

we can conclude that the quasi stationary density $\hat{p}_1(z)$ in fact is the Yaglom-limit of the process and $e^{-\lambda _1 t}$ is the related survival function. Note that no other function $e^{-\lambda _i t},\,i\ne 1$ can be used as survival function even if the related normalized function $\bar{p}_i$ would qualify as a quasi stationary density, because the related limit would be infinity. $\square$

**Fig. 4:**
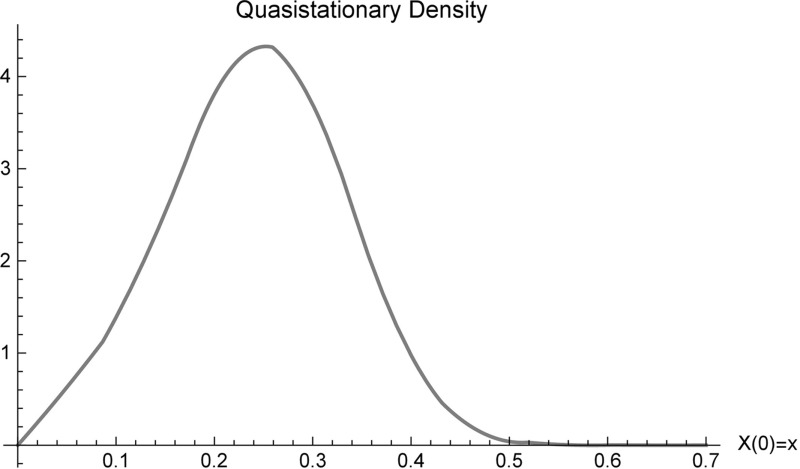
Quasi-stationary density (and Yaglom-limit) of the process with $\beta =1.1,\,\gamma =0.8$ and $N=100$, see also the first line of Fig. 3. The related eigenvalue is given by $\lambda _1\approx 0.00293006$

Using numerical methods (see e.g. Ishikawa 2007) we calculated eigenvalues and eigenfunctions for the three processes shown in Fig. 3. Only for the process with $\beta =1.1,\,\gamma =0.8$ and $N=100$ (first line of Fig. 3) the smallest eigenvalue has an eigenfunction that can be normalized to a density $\bar{p}_1$. So in this case the quasi stationary density $\bar{p}_1$, which is shown in Fig. 4, is also the Yaglom-limit. The value of the smallest eigenvalue, $\lambda _1\approx 0.00293006$ shows slow decay to the disease free distribution.

The smallest eigenvalue for the models with $\beta =0.8,\,\gamma =1.1$, $N=100$ and $\beta =\gamma =0.9,\,N=100$ are $\lambda _1\approx 0.929041$, respectively $\lambda _1\approx 0.100599$. Hence in both cases we have quick decay to the disease free distribution.

### Time until absorption

In order to complement the analysis of quasi-stationarity, we analyze the expected time until absorption at zero (mean exit time, mean first passage times) when starting at *x*. If the random variable $\tau$ denotes the first time at which the process hits zero, i.e. $\tau =\inf \left\{ t:\,Z(t)=0\right\}$ then the expected absorption time is given by $T(z)={\mathbb {E}}\left[ \tau |X(0)=z\right]$ and the expectation *T*(*z*) fulfills the differential equation [see e.g. Gardiner (2009) section 5.5.2, for another application to an epidemiological model see van Herwaarden (1997)]

$$\begin{aligned} A(z)\frac{\partial T(z)}{\partial z}+\frac{1}{2}B(z)\frac{\partial ^{2}T(z)}{\partial z^{2}}+1=0 \end{aligned}$$

with boundary conditions $\frac{\partial T}{\partial z}(1+\gamma /\beta )=0$ and $T(0)=0$. With

$$\begin{aligned} \psi (z)=\exp \left[ 2\int _{0}^{z}A(y)/B(y)\,dy\right] =e^{2Nz}\left( \frac{\beta \cdot (1-z)+\gamma }{\beta +\gamma }\right) ^{\frac{4\gamma N}{\beta }} \end{aligned}$$

the solution can be written as

$$\begin{aligned} T(z)=2\int _{0}^{z}\frac{1}{\psi (y)}\int _{y}^{1+\gamma /\beta }\frac{\psi (x)}{B(x)}dx\,dy. \end{aligned}$$

**Fig. 5:**
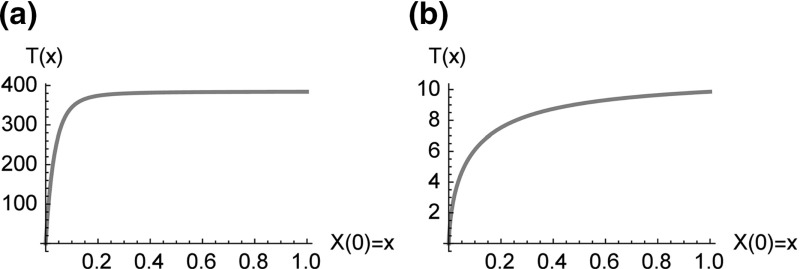
Expected time until absorption at zero as a function of the starting value. **a** Slow extinction of the disease: model $\beta =1.1,\,\gamma =0.8$ and $N=100$. **b** Quick extinction: The model $\beta =0.8,\,\gamma =1.1$ and N = 100

Figure 5 shows the expected time to absorption at zero as a function of the starting value for two of the models, already depicted in Fig. 3.

## Taking decisions on treatment intensity

In the following we assume that a decision maker is able to take a decision on the size of the recovery rate $\gamma$. This can be e.g. related to investment into treatment capacity or treatment efficiency. Increasing the recovery rate leads to costs, while on the other hand infected individuals also create costs like e.g. the costs of lost working time. The aim is then to find an optimal size of the recovery rate in terms of the related costs.

We assume that the decision has to be taken “here and now” at the beginning of the (infinite) planning horizon, and that it cannot be reconsidered later. Moreover we neglect the effect of interest yields (or alternatively we may assume that the transition to the stationary distribution is quick). For a stochastic control model related to the dynamics (5) (with $h=0$) over a finite time horizon with discounting see Grandits et al. (2016).

We start with redefining the recovery rate as

$$\begin{aligned} \gamma =\gamma _0+u, \end{aligned}$$

where $\gamma _0$ is the “natural” recovery rate without additional measures, and $u\ge 0$ denotes the additional size of the treatment effect, chosen by the decision maker. It is assumed that $\gamma =\gamma _0$ fulfills condition (10), which means that we have a choice between accepting some stationary distribution $p_s(x)$ and full extinction of the disease, when choosing *u*. For fixed *u* we denote the process described by (5) by $X_u(t)$ and the related stationary density by $p_s(x;u)$.

In order to model costs and the objective function, we assume that increasing the natural recovery rate by an additional amount of *u* results in costs $K(X_{u}(t),u)$ at time *t*.

### The model with external parameter noise and saturated incidence

When analyzing the model with saturated incidence (5), we look directly at the stationary distribution (9), respectively $\delta (x)$, depending on the parameter values. Clearly this is a simplification, but also has the advantage that optimization can be done by standard numerical methods. In particular we use the ergodicity of the process (see the discussion after Fact 1) and aim at minimizing the (long run) expected average costs per unit time *EAC*:

When (10) is fulfilled for nonnegative *u*, i.e.

$$\begin{aligned} 0\le u<N\beta -\frac{1}{2}N^{2}\sigma ^{2}-\gamma _{0}, \end{aligned}$$

then we can use the ergodicity properties of the process $X_{\gamma }(t)$, which leads to

35 $$\begin{aligned} EAC(u)= & {} EAC_{1}(u)=\lim _{T\rightarrow \infty }\frac{1}{T}{\mathbb {E}}\left[ \int _{0}^{T}K(X_{u}(t),u)\right] ={\mathbb {E}}_s\left[ X_{u}\right] \nonumber \\= & {} \int _{0}^{N}K(x,u)\,p_{s}(x;u)\,dx. \end{aligned}$$

If (10) is violated, i.e. if

$$\begin{aligned} u\ge N\beta -\frac{1}{2}N^{2}\sigma ^{2}-\gamma _{0} \end{aligned}$$

then we have

36 $$\begin{aligned} EAC(u)= & {} EAC_{2}(u)=\lim _{T\rightarrow \infty }\frac{1}{T}{\mathbb {E}}\left[ \int _{0}^{T}K(X_{u}(t),u)\right] \nonumber \\= & {} \int _{0}^{N}K(x,u)\,\delta (x)\,dx=K(0,u). \end{aligned}$$

Clearly the cost function *K* has to be chosen such that the expectation at the right hand side of (35) is finite. This is the case for a reasonable class of of cost functions.

#### Proposition 4

Let (for any $u\in I=[0,N\beta -\frac{1}{2}N^{2}\sigma ^{2}-\gamma _{0}]$) the cost function *K*(*x*, *u*) be continuous almost everywhere, monotonically increasing in *x* for $x\in [0,N]$ and bounded on [0, *N*]. Then we have

$$\begin{aligned} {\mathbb {E}}[K(X_{u},u)]=\int _{0}^{N}\left| K(x,u)\right| \,p_{s}(x;u)\,dx<\infty , \end{aligned}$$

Moreover, if $X_{u}$ has stationary distribution $p_{s}(x;u)$ as defined in Proposition 1, then all noncentral moments ${\mathbb {E}}\left[ (X_{u})^{k}\right] ,\,k\in {\mathbb {N}}$ (and consequently also all central moments) are finite.

#### Proof

Using monotonicity and boundedness we get

$$\begin{aligned} \int _{0}^{N}\left| K(x,u)\right| \,p_{s}(x)\,dx\le \left| K(N,u)\right| <\infty . \end{aligned}$$

The functions $K(x,u)=x^{k},\,k\in {\mathbb {N}}$ fulfills the assumptions of the corollary, which implies existence of moments. $\square$

In addition to the requirements of Proposition 4, it is reasonable to use functions that are convex in *u* and *x*, although this does not guarantee convexity of the objective function (in *u* which is an argument of the cost function but also a parameter of the stationary density $p_{s}$).

The disease becomes extincted already for $u=N\beta -\frac{1}{2}N^{2}\sigma ^{2}-\gamma _{0}$ and any higher value of *u* would just lead to extinction at higher expected average costs. Taking this into account, one has to decide whether it is cheaper to accept some endemic distribution of the disease or to erase it fully.

Taking into account the cases (35) and (36) and denoting

$$\begin{aligned} \varGamma = \left\{ u\in {\mathbb {R}}:\,0\le u \le N\beta -\frac{1}{2}N^{2}\sigma ^{2}-\gamma _{0}\right\} . \end{aligned}$$

the optimization problem (minimizing the expected average costs) can be written as

37 $$\begin{aligned} \min _{u\in \varGamma }\,EAC(u). \end{aligned}$$

Three cases have to be compared for finding the minimizer: the lower bound $u=0$, the upper bound $u=N\beta -\frac{1}{2}N^{2}\sigma ^{2}-\gamma _{0}$ and a possible inner solution $0<u<N\beta -\frac{1}{2}N^{2}\sigma ^{2}-\gamma _{0}$.

Unfortunately it is not possible to guarantee any nice properties like convexity for problem (37). Moreover, an analytic treatment is not possible. The difficulties come from the fact that there is no analytic solution known for the integrals of the stationary distribution $p_s(x;u)$ defined in (9). In particular, even the normalizing constant *C*(*u*) has to be calculated numerically for possible values of *u*. As will be shown in an example below, the usual case comes down to a comparison between $u=0$ and $u=N\beta -\frac{1}{2}N^{2}\sigma ^{2}-\gamma _{0}$.

### A note on the model with scaled additive noise

No stationary distribution exists for the model (17) as pointed out above. Nevertheless, the optimization approach described in the previous subsection can be extended in a simple way in order to deal with this situation.

Assuming that the limiting quasi stationary distribution quickly dominates the decomposition (33), which is in particular the case if the eigenvalue $\lambda _1$ is sufficiently small compared to the other eigenvalues, we focus on the analysis of the quasi stationary distribution instead of the stationary distribution.

In such a situation we may neglect the second part, i.e. asymptotically we have

$$\begin{aligned} p(z,t)\backsim \bar{p}_1(z)e^{-\lambda _1 t}. \end{aligned}$$

The main part of expected costs then (again neglecting interest) can be written as

$$\begin{aligned} EC(u)= & {} \int _{0}^{\infty }\int _0^N K(x,u)p(x,t)\,dx dt=\int _{0}^{\infty }\left[ \int _0^N K(x,u)\bar{p}_1(x)\,dx\right] e^{-\lambda _1(u) t}\, dt\\= & {} \frac{{\mathbb {E}}_{Q(u)0}\left[ K(X,u) \right] }{\lambda _1(u)}, \end{aligned}$$

where ${\mathbb {E}}_{Q(u)}\left[ K(X,u) \right] =\int _{0}^{\infty }K(x,u)\bar{p}_1(z)\,dt$ is the expectation of the quasi stationary distribution with density $\bar{p}_1(z)$. The notation *Q*(*u*) emphasizes the fact that the stationary density depends on *u*—in particular it involves a normalizing factor that depends also on *u*.

Maximizing *EC*(*u*) with respect to the constraint $u\ge 0$ is a fractional optimization problem. Again, the exact properties (e.g. possible pseudoconcavity of the objectve) of this problem is unknown.

### A numerical example

For a numerical example we use the simple cost function

38 $$\begin{aligned} K(x,u)=c_0 u + c_1 x u + c_2 x, \end{aligned}$$

where $c_2$ denotes cost per infected individual (e.g. costs for sickness leaves or the value of lost labour), $c_0$ is cost for an increase of the recovery rate (e.g. investment costs for additional treatment capacity, depreciation) and $c_1$ denotes treatment costs per individual, time unit and level of recovery rate. Clearly, in a more refined model, the relation between treatment intensity and the recovery rate could be modeled by a nonlinear function.

**Fig. 6:**
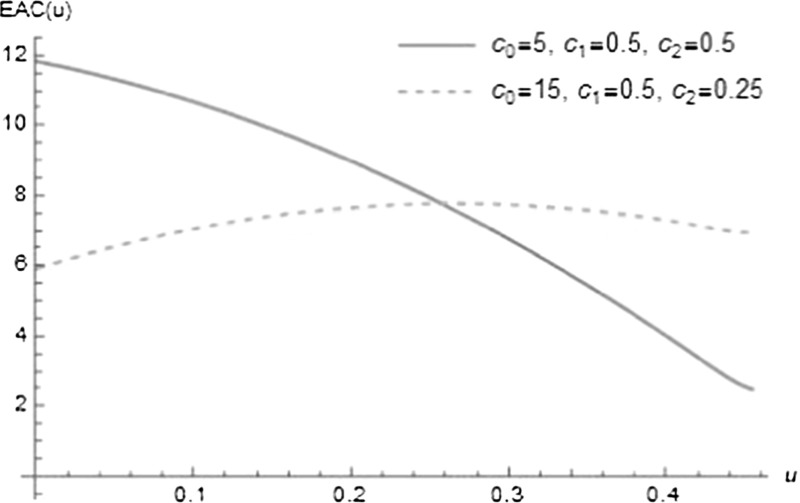
Expected average costs, depending on the additional recovery rate *u*, for the model $N=100,\,\beta =0.013,\,\gamma =1,\,h=0.05,\,\sigma =0.005$ and two cost structures (see the legend)

This cost function is applied to the process with parameter values $N=100,\,\beta =0.013,\,\gamma =1,\,h=0.05$ and $\sigma =0.005$, which was analyzed earlier, see Fig. 2. The resulting expected average costs for two cost structures ($c_0=5, c_1=0.5, c_2=0.5$ and $c_0=15, c_1=0.5, c_2=0.25$) can be seen in Fig. 6. In this case the optimal decisions are at the boundary of the feasible region: in the first case it is optimal to use the full capacity of treatment and to extinct the disease. In the second case treatment intensity is too expensive and it is optimal to stay at the natural level of recovery. Empirically, this pattern seems to be valid also for other parameter values for the process and the cost function (38).

## Conclusions

We analyzed two kinds of stochastic SIS models in order to derive some of their basic properties from the related Fokker–Planck equations. The first type of model was recently presented in Chen and Kang (2014): a model with saturated incidence, augmented with random disturbances of the strength of infection $\beta$. It turns out that under a suitable condition (10) on the risk adjusted basic reproduction number there is a well behaved stationary density related to the process of infected individuals. Besides the pure question of existence, it is possible to calculate the stationary density explicitly. Moreover, the shape of the density, in particular its behaviour at zero, was analyzed further. If the basic condition (10) is violated, the only possible (generalized) density is given by the Dirac delta function at zero, which indicates that the process of infected individuals converges (almost surely) to zero. Briefly worded, the stochastic model here shows weak convergence to either a stochastic endemic equilibrium or to extinction. In principle this resembles the two possible cases—endemic equilibrium and extinction—for deterministic versions of the SIS model with saturated incidence.

The second type of model treated in this paper can be derived from a continuous time Markov chain approach by applying the Kramers–Moyal approximation. No stationary density except the Dirac delta function exists in this case. Convergence of the process of infected to zero—extinction of the disease—happens almost surely and for each time $t>0$ there is a positive probability for extinction of the disease. This behaviour is quite different from the case with extinction in the first model. In fact, the stochastic diffusion approximation inherits this property from the underlying Markov chain. Still it is possible to analyze quasi-stationary densities and the Yaglom-limit and to calculate quantities like the expected time until extinction.

Finally, we analyzed the decision problem of choosing the optimal level of the treatment intensity (recovery rate) and gave some numerical example.

Many open question remain for further research. The overall approach can be applied to higher dimensional models with more than two relevant classes of individuals. Furthermore, several other approximation methods can be compared to the Kramers–Moyal approximation used for the second model in the present paper. Finally, the optimization approach here was static so far. A genuinely dynamic extension of the treatment decision in terms of stochastic optimal control will definitely be an interesting topic for further studies.

## Footnotes

1 Note that there exist controversies and even conflicting nomenclature on the exact structure of the transmission term, in particular regarding the role of the parameter $\beta$. As a simplified overview, we use a force of infection of $\beta X(t)$. This specification is often referred to as “pseudo-mass action” or “density dependent”. Here, the the number of contacts depends (implicitly) on the population size. The most important alternative is given by the force of infection $\beta X(t)/N$, where the force is normalized, such that the number of contacts does not depend on the population size. This specification is often referred to as “mass action” or “frequency dependent”. In some sense the frequency dependent specification is more natural and leads to simpler results, because e.g. the basic reproduction number (3) and related results for the stochastic models (compare footnote 3 below) then do not depend on the population size *N*. On the other hand, the exact type of specification is important only in more complicated models (including e.g. birth and death) where *N* depends on time. Basically, *N* is just a constant here, and one can redefine $\beta =\hat{\beta }/N$ for some parameter $\hat{\beta }$, related to the frequency dependent specification. Therefore we stick to the pseudo-mass specification, which is used in the majority of papers we cite on stochastic formulations of SIS models.

2 Recall that a measure *P* is called ergodic if $P(A)\in \{0,1\}$ for every measurable set *A* which is invariant under the system dynamics. In our case the invariant sets are given by $\{0\}$ and (0, *N*).

3 Note that (10) is reduced to the simpler $R_1=\frac{\hat{\beta }-\frac{1}{2} \hat{\sigma }^{2}}{\gamma }$, if $\beta$ is defined frequency dependent with parameter $\hat{\beta }$ (compare footnote 1) and $\sigma =\hat{\sigma }/N$.

## References

[CR1] Abad FX, Pinto RM, Bosch A (1994). Survival of enteric viruses on environmental fomites. Appl Environ Microbiol.

[CR2] Albina E (1997). Epidemiology of porcine reproductive and respiratory syndrome (PRRS): an overview. Vet Microbiol.

[CR3] Altay S, Schmock U (2013) Lecture notes on the Yamada–Watanabe condition for the pathwise uniqueness of solutions of certain stochastic differential equations. Lecture Notes. http://www.fam.tuwien.ac.at/~schmock/notes/Yamada-Watanabe.pdf

[CR4] Barbour AD (1976). Quasi-stationary distributions in Markov population processes. Adv Appl Probab.

[CR5] Behrens JP, Gaulkins D Aand, Tragler G, Feichtinger G (2002). Why present-oriented societies undergo cycles of drug epidemics. J Econ Dyn Control.

[CR6] Bjornstad ON, Finkenstädt BF, Grenfell BT (2002). Dynamics of measles epidemics: estimating scaling of transmission rates using a time series SIR model. Ecol Monogr.

[CR7] Brauer F, Allen LJS, Van den Driessche P, Wu J (2008). Mathematical epidemiology.

[CR8] Cacio E, Cohn SE, Spigler R (2012). Numerical treatment of degenerate diffusion equations via Feller’s boundary classification, and applications. Numer Methods Partial Differ Equ.

[CR9] Capasso V, Serio G (1978). A generalization of the Kermack–McKendrick deterministic epidemic model. Math Biosci.

[CR10] Chen C, Kang Y (2014). Dynamics of stochastic SIS epidemic model with saturated incidence. Abstr Appl Anal.

[CR11] Collet P, Martinez S, Martin JS (2012). Quasi-Stationary distributions: Markov chains, diffusions and dynamical systems.

[CR12] Dexter N (2003). Stochastic models of foot and mouth disease in feral pigs in the Australian semi-arid rangelands. J Appl Ecol.

[CR13] Diekmann O, Heesterbeck H, Britton T (2013). Mathematical tools for understanding infectious disease dynamics.

[CR14] Feller W (1952). The parabolic differential equations and the associated semi-groups of transformations. Ann Math.

[CR15] Fleming WH, Rishel RW (1975). Deterministic and stochastic optimal control.

[CR16] Fuchs C (2013). Inference for diffusion processes with applications in life science.

[CR17] Gardiner C (2009). Stochastic methods—a handbook for the natural and social sciences.

[CR18] Gould JP (1970). Microeconomic foundations of employment and inflation theory.

[CR19] Grandits P, Kovacevic RM, Veliov V (2016) Optimal control and the value of information for a stochastic epidemiological SIS-model. Research Report 2016-14, ORCOS, TU, Wien

[CR20] Gray A, Greenhalgh D, Hu L, Mao X, Pan J (2011). A stochastic differential equation SIS epidemic model. SIAM J Appl Math.

[CR21] van Herwaarden OA (1997). Stochastic epidemics: the probability of extinction of an infectuous disease at the end of a major outbreak. J Math Biol.

[CR22] Hethcote HW, Yorke JA (1984). Gonorrhea transmission dynamics and control. Lecture Notes in Biomathematics.

[CR23] Ishikawa H (2007). Numerical methods for the eigenvalue determination of second-order ordinary differential equations. J Comput Appl Math.

[CR24] Kallenberg O (2002). Foundations of modern probability.

[CR25] Kandhway K, Kuri J (2014). How to run a campaign: optimal control of SIS and SIR information epidemics. Appl Math Comput.

[CR26] Keeling MJ, Grenfell BT (1999). Stochastic dynamics and a power law for measles variablility. Philos Trans R Soc B.

[CR27] Keeling MJ, Rohani P (2008). Modeling infectuous disease in humans and animals.

[CR28] Keeling MJ, Ross JV (2008). On methods for studying stochastic disease dynamics. J R Soc Interface.

[CR29] Kryscio RJ, Lefévre C (1989). On the extinction of the S-I-S stochastic logistic epidemic. J Appl Probab.

[CR30] Lahrouz A, Omari L, Settati A, Belmaati A (2015). Comparison of deterministic and stochastic SIRS epidemic model with saturating incidence and immigration. Arab J Math.

[CR31] Lin Y, Jiang D, Wang S (2014). Stationary distribution of a stochastic SIS epidemic model with vaccination. Phys A.

[CR32] Mahajan VE, Muller E, Bass FM (1993). Marketing.

[CR33] McKane AJ, Newman TJ (2005). Predator–prey cycles from resonant amplification of demographic stochasticity. Phys Rev Lett.

[CR34] Meleard S (2012). Quasi-stationary distributions and population processes. Probab Surv.

[CR35] Murray JD (1989). Mathematical biology.

[CR36] Nasell I (2011). Extinction and quasi-stationary in the stochastic logistic SIS model.

[CR37] Ovaskainen O, Meerson B (2010). Stochastic models of population extinction. Trends Ecol Evol.

[CR38] Risken H (1989). The Fokker–Planck equation—methods of solution and applications.

[CR39] Roberts MG, Saha AK (1999). The asymptotic behaviour of a logistic epidemic model with stochastic disease transmission. Appl Math Lett.

[CR40] Rohani P, Keeling MJ, Grenfell BT (2002). The interplay between determinism and stochasticity in childhood diseases. Am Nat.

[CR41] Yamada T, Watanabe S (1971). On the uniqueness of solutions of stochastic differential equations. J Math Kyoto Univ.

[CR42] Zhang Z, Chen D (2013). A new criterion on existence and uniqueness of stationary distribution for diffusion processes. Adv Differ Equ.

